# The potential association between PARP14 and the SARS-CoV-2 infection (COVID-19)

**DOI:** 10.4155/fmc-2020-0226

**Published:** 2021-01-20

**Authors:** Amanda L Tauber, Stephanie S Schweiker, Stephan M Levonis

**Affiliations:** ^1^Faculty of Health Sciences & Medicine, Bond University, Gold Coast, Queensland 4229, Australia

**Keywords:** ARTD8, coronavirus, COVID-19, immunity, PARP14, SARS-CoV-2, viral

## Abstract

Understanding the potential association between the poly (ADP-ribose) polymerase member 14 (PARP14) and the severe acute respiratory syndrome coronavirus 2 (SARS-CoV-2) may aid in understanding the host immunopathological response to the virus. PARP14 has an emerging role in viral infections, and this article considers its potential mechanisms for action in either a pro- or anti-viral manner. It is evident that more experimental work is required; however, PARP14 appears vital in controlling the interferon response to the SARS-CoV-2 infection and has potential roles in balancing the proinflammatory cytokines of the cytokine storm. Furthermore, the SARS-CoV-2 macrodomain can prevent the PARP14-mediated antiviral response, suggesting a more complex relationship between PARP14 activity and SARS-CoV-2 infections.

## Tweetable abstract

Although the enzyme PARP14 has been associated with negative outcomes in cancers and allergic airway diseases, it may in fact aid in equipping the immune response against the novel 2019 coronavirus.

The year 2020 was dominated by the effects of the 2019 coronavirus disease (COVID-19), and consequently the race to understand its mechanism of infection and uncover effective treatment options has taken precedence for many research groups. Patients with severe COVID-19 often display a unique pattern of immune dysregulation, causing us to consider the potential involvement of the poly (ADP-ribose) polymerase member 14 (PARP14, also known as ARTD8, BAL2 or CoaSt6). This posttranslational modifier enzyme is most commonly known for its involvement in promoting tumorigenesis and allergic airway diseases [1]. However, recently it has been found to have an emerging role in viral infection [1]. In this article we aim to consider the biochemical mechanism of the severe acute respiratory syndrome coronavirus 2 (SARS-CoV-2) infection and whether PARP14 is known (or is likely) to play a role. If it does have a role, would PARP14 inhibition aid in the treatment of COVID-19, or would it promote viral progression?

## SARS-CoV-2 infection

COVID-19 is a disease caused by SARS-CoV-2, a member of the Coronaviridae family. SARS-CoV-2 is an enveloped, positive-sense single-stranded RNA virus of approximately 100 nm in circular diameter and is covered in large spiked glycoproteins across the membrane surface [2]. During infection, SARS-CoV-2 utilizes ACE2 as a binding receptor and TMPRSS2 as a cofactor to activate its attachment proteins, leading to its internalization and replication within the host cells [3]. As ACE2 is widely expressed on the nasal and oropharyngeal epithelium, these areas within the upper respiratory tract are the first site of viral infection [4]. In many cases of COVID-19, patients only exhibit mild symptoms; however, a considerable population will develop far more severe symptoms, requiring intensive medical care and placing them at a higher risk of death [4].

Although the exact pathogenesis of COVID-19 and the SARS-CoV-2 infection is still being elucidated, the virus is observed to cause injury to the immune system [4]. As PARP14 has a well-established role in immune regulation, including its dysregulation of the Th2 immune response during allergic airway diseases, this route presented itself as a potential means of PARP14 involvement in COVID-19 pathogenesis. The SARS-CoV-2 immune injury is closely linked to the development of the systemic inflammatory response syndrome, and if left untreated can culminate in multiple organ failure [2]. This has also been noted in other coronaviruses such as SARS-CoV and MERS-CoV [2]. While a rapid and effective immune response is desirable during infection, an excessive response can itself cause damage. Part of the systemic inflammatory response syndrome observed in SARS-CoV-2 is the ‘cytokine storm’, a mass secretion of cytokines (e.g., IL-1β, IL-1RA IL-6, IL-7, IL-8), and the severity of this cytokine storm is closely linked to disease severity and mortality [3,5,6]. It is suggested that an early anti-inflammatory intervention can help prevent immune damage and thus reduce any potential injury to the nervous system [6].

Upstream from the SARS-CoV-2 cytokine storm, macrophages, dendritic cells and monocytes are activated and release IL-6, which binds to the IL-6R, activating intracellular signal transducers such as the JAK/STAT pathway (primarily via STAT1 and STAT3 isoforms), JNK proinflammatory pathway, MAPK pathway and PI3K pathway [7]. During SARS-CoV-2 infection, the high activation of these pathways causes the release of inflammatory cytokines which, in excess, become the cytokine storm [5]. IL-6 is further promoted by TLR4 from the NFKB pathway, steering the viral progression toward an excessive activation of the innate immune response, as observed through the inflammatory induced damage to the pulmonary interstitial arteriolar walls [3]. Many COVID-19 investigations focus on IL-6, claiming it is the prime suspect for inducing the proinflammatory response within the body and correlating it positively with the severity of COVID-19 symptoms [5,6]. Multiple targets have been suggested to halt or suppress this cytokine storm, including the use of tocilizumab – an anti-IL-6R monoclonal antibody which blocks the membrane receptor binding of IL-6 – or anakinra, an IL-1 receptor antagonist [5]. PI3K inhibitors have also showed promise in idiopathic pulmonary fibrosis, along with inhibition of the JNK family impairing the synthesis of H5N1 viral RNA [8]. Future proposals include inhibitors of the JAK-STAT pathway (e.g., baricitinib, which was proposed using artificial intelligence algorithms) [9].

Furthermore, several SARS-CoVs have been shown both in isolation and *in vivo* to suppress the cellular interferon response, potentially enhancing the virulence of SARS-CoV. However, the presence of interferon is seen to have a dual role, as demonstrated by Fehr *et al.*; if CoV-infected murine models lacking IFN-1 signaling (*IFNAR*^-/-^) received exogenous IFN-1 prior to the peak virus replication, the mice were completely protected from the disease [10]. These results support the use of interferons as a treatment option for SARS-CoVs and highlight the importance of interferon production in protection against the virus [10]. However, it was also seen that without the initial administration, interferons were rapidly produced later in the infection, recruiting inflammatory monocytes to the lung and causing the production of additional proinflammatory cytokines, eventually leading to lethality. The authors restated that they were unable to confidently predict the role of interferon in *IFNAR*^-/-^ due to this apparent dual role [10]. Other studies report that when interferons are coadministered *in vitro* with the antiviral ribavirin, their activity is increased compared with administration of interferons alone [11].

Another aspect to consider regarding the virulence of SARS-CoV-2 and its potential association with the macrodomain-containing PARP14 is the SARS-CoV macrodomain. Located within the transmembrane nonstructural protein 3 (nsp3), this macrodomain is highly conserved across the Coronaviridae subfamily and is suggested to be vital for the virulence of the virus [10]. Many other viruses also encode a macrodomain which acts to bind and hydrolyze ADPr from proteins, and in SARS-CoVs is regarded as essential for its ADP-ribose-1′-phosphatase activity *in vitro* [10]. Studies by Fehr *et al.* in 2016 mutated the D1022A, N1040A, H1045A or G1130V residues of the mouse-adapted virus model MA15 to either limit or eliminate the ADP-ribose-1′-phosphatase activity [10]. They found that the SARS-CoV macrodomain suppressed the early interferon and proinflammatory cytokine response for the host immune system, consequentially promoting lung edema and lethality in the infected mice. However, in mice exposed to the virus that lacked the catalytic activity of the macrodomain (N1040A mutation), the virus induced significantly elevated expression of interferon-stimulated genes and other cytokines. Furthermore, when mice were coinfected with both wild-type and N1040A-mutated viruses, they still had better outcomes and increased interferon and proinflammatory cytokine expression than those infected with the wild-type virus alone [10]. The critical nature of the macrodomain has also been observed using the prototypical coronavirus model, mouse hepatitis virus strain JHMV (MHV), as well as the sindbis viruses, in which mutations in the macrodomain render the virus unable to cause severe hepatitis and encephalitis [10]. These results collectively demonstrate the prominent role the viral macrodomain has in promoting virulence. The Fehr *et al.* study also noted that, although the macrodomain was associated with dephosphorylating ADR-1″-phosphate to ADR, this intermediate had never been detected in a coronavirus infection, suggesting that the macrodomain was more likely involved in deMARylating or dePARylating target proteins [10]. The specific molecular targets of the coronavirus macrodomains still need to be explored; however, given this de-MAR/PAR activity, it is likely that the coronavirus macrodomain has some opposing role to ADP ribosylation, and likely the PARP family.

## PARP14’s potential role in SARS-CoV-2 infection

Although the above is not intended as a comprehensive review of the SARS-CoV-2 mechanism of action, it does highlight some areas in which PARP14 may have some involvement; in particular, SARS-CoV-2’s association with inflammatory cytokines and the interferon response and its potential to reverse the MAR activity of PARP14. As previously mentioned, inflammatory cytokines are a key component of the cytokine storm exhibited in the SARS-CoV-2 infection, in particular those related to STAT1/STAT3 activation and pro-inflammatory cytokines. This expression is in opposition to those associated with PARP14 activity: PARP14 is involved in promoting the STAT6-dependent transcription of the Th2 immune response, whereas SARS-CoV-2 promotion of STAT1 transcription is associated with a Th1 response [1,12], and the predominant cytokine of PARP14 activation is IL-4 compared with SARS-CoV infections with IL-6. This opposition suggests that rather than promoting the release of inflammatory cytokines associated with SARS-CoV-2, PARP14 (if involved) may play a role in the host response to counteract the immune imbalance. A 2020 correspondence by Webb and Saad also suggested that PARP14 may play a role in the host response to SARS-CoV-2 infection by counteracting skewing of the Th1:Th2 cytokine ratio [12]. They support this theory in part due to the 32% structural homology between the macrodomain of PARP14 and SARS-CoV-2, stating that the similarity may be caused by the SARS-CoV macrodomain coevolving with ADPr proteins to counter their activity [12]. However, this potential role of PARP14 counteracting the inflammatory cytokines observed in SARS-CoV-2 infection is purely speculative and will need experimental validation.

Another potential mode of action for PARP14 in SARS-CoV-2 infection is via the interferon response. A study by Grunewald *et al.* in 2019 suggested that there was strong evidence showing that PARP14 ADP ribosylation (ADPr) is involved in the stimulation of IFN-1. This is promising evidence for PARP14’s role in host defense against SARS-CoV-2; IFN-1 treatment is often proposed as a candidate treatment during viral infection [13]. The sensitivity of SARS-CoV (using the human coronavirus 229E) to IFN-α treatment was also increased when the coronavirus macrodomain was attenuated [12]. Furthermore, an uncontrolled, exploratory study showed that for patients with COVID-19, IFN-α2b therapy appeared to shorten the duration of viral shedding, accelerating viral clearance from the respiratory tract and reducing the concentration of IL-6 [14]. A more controlled study would need to take place to better validate these conclusions.

This association between PARP14 and its potential interferon response is further explored when investigating the coronavirus macrodomain. There is preliminary evidence to suggest that the SARS-CoV-2 macrodomain interacts with PARP14. As previously mentioned, coronaviruses with mutated or absent macrodomains were associated with reduced viral loads, as well as increased sensitivity to IFN-1 treatment in cell culture, suggesting that the coronavirus macrodomain counters antiviral activities [10,15]. A key PARP14 and coronavirus study by Gruenwald *et al.* firstly looked at the effect of pan-PARP inhibitors (3-aminobenzamide and XAV-939) at high concentrations against the murine hepatitis virus (MHV), finding a resulting decrease in interferon production which was not observed in the wild-type (WT) virus [15]. They inferred that the ADP ribosyltransferase activity was necessary for PARP14’s antiviral activity, as the pan-PARP inhibitors target the catalytic domain; however, this is limited as the pan-PARP inhibitors target all 17 PARP enzymes [15]. To look more directly at PARP14, they used the inhibitor **8k**, which is selective toward PARP14 when compared with PARP1 (with IC_50_ values of 0.78 and 19 μM respectively) and is slightly more selective over PARP10 at 1.4 μM [16]. Using **8k** in combination with PARP14^−/−^ bone marrow-derived macrophages (BMDMs), along with human PARP14 knockout (KO) A549 and normal human dermal fibroblast (NHDF) cells, the authors showed that PARP14 was required to induce the heightened IFN-1 production during coronavirus infection. This was consistent with other studies detailing PARP14’s role in IFN-1 induction following lipopolysaccharide (LPS) stimulation of RAW 264.7 cells and BMDMs [15].

The study also details the creation of the recombinant virus N1347A, which contains an alanine mutation within the macrodomain that effectively removes the ADP hydrolase activity of MHV macrodomains. Within murine models, this virus was shown to replicate poorly and was not disease-causing, highlighting the importance of the viral macrodomain to counter antiviral activities [15]. As in the MHV studies, the authors used siRNA knockdown with the PARP14 inhibitor **8k** to show that although **8k** did not affect the cell viability, metabolism, or global cellular PARylation, it restored the replication of the N1347A virus in BMDMs significantly [15], thereby supporting the role of PARP14 blocking N1347A MHV replication [15]. Other PARPs may also be involved in the antiviral activity, as the pan-PARP inhibitors were able to reduce N1347A IFN-1 levels much more effectively than the partially specific PARP14 inhibitor [15]. Together, this study showed that PARP14 was required to inhibit the replication of the mutated coronavirus and demonstrated its importance in interferon expression. Gruenwald *et al.* were not the only ones to start investigating PARP14’s emerging role in viral defense; among a few preliminary studies, an early 2018 paper investigated the previously uncharacterized association PARP14 has with the regulation of IFN-β response in murine macrophages [17]. Following endotoxin stimulation, PARP14 was shown to bind to a small group of specific interferon-stimulated gene-encoded proteins, enabling their nuclear accumulation. This was further reinforced as following the loss of PARP14, the transcription of IRF3-regulated primary response genes was attenuated, resulting in the reduction of IFN-β and the activation of secondary antiviral response genes [17], again validating PARP14’s emerging role in the stimulation and regulation of type 1 interferons during a viral attack.

## PARP14 inhibitors

There are three potential pathways in which PARP14 may have a role in aiding the host immune system against SARS-CoV infections; however, all require further experimental validation. A key aspect of investigating the potential role of PARP14 is the availability of potent and selective PARP14 inhibitors. Currently, there are no clinically available PARP14 inhibitors, but there are some promising small-molecule lead compounds. As discussed earlier, one study utilized the PARP14 inhibitor lead compound **8k**, which exhibited sub-micromolar potency against PARP14’s catalytic domain while also showing the highest selectivity over PARP1 [16]. However, this compound is limited in its ability to be classified as a selective PARP inhibitor, because its potency has not been assessed against all PARP enzymes. Another promising lead has been **H10**, as proposed by Peng *et al*., which was found to inhibit PARP14 activity at an IC_50_ of 0.49 ± 0.07 μM *in vitro* [18]. **H10** operates as a bidentate inhibitor, targeting both the conserved nicotinamide binding site and the less conserved adenine binding site within PARP14’s catalytic domain. To date, **H10** is the most potent PARP14 inhibitor; however, like **8k**, it requires further assessment against the other PARP family members before we can be confident in its selectivity. Suggestions by Grundewald *et al.* imply that it is PARP14’s catalytic domain which is responsible for its potential antiviral action. However, it would be of interest to investigate whether PARP14’s macrodomain also plays a role. This aligns nicely with the current studies that are targeting the macro2 domain of PARP14, in the hope of achieving greater selectivity toward PARP14 over the other PARPs. The most effective macrodomain inhibitors target the macro2 domain and include the carbazole **108**, which possesses sub-micromolar activity with an IC_50_ value of 0.66 ± 0.03 μM [19].

Furthermore, whilst PARP14 appears to be involved in aiding the host defense against SARS coronaviruses, the earlier DNA-dependent poly (ADP-ribose) polymerases (PARP1–3) are speculated to have an opposing role. A review by Curtin *et al.* proposes a clinical investigation of DNA-dependent PARP inhibitors against COVID-19 [4], highlighting that PARP1 and PARP2 actively increase and prolong inflammation and that the use of PARP inhibitors can limit this tissue damage, as well as decreasing the levels of proinflammatory cytokines [4]. They continue to pitch a COVID-19 clinical trial using the clinically approved (for primarily *BRCA*-mutated cancers) PARP inhibitors, justifying that the doses would likely not need to be as high as those used in oncological applications [4]. Overall, it is a very well-done study that condenses the evidence for the use of DNA-dependent PARP inhibitors in coronavirus treatment.

## Conclusion & future perspective

Our original aim was to investigate whether PARP14 plays a role in the pathology of COVID-19 and whether a PARP14 inhibitor would be useful as a treatment option. Overall, there needs to be more experimental evidence to support PARP14’s potential role in host defense against SARS-CoV-2. However, we have identified three potential modes of action, including PARP14 counterbalancing the proinflammatory cytokines of the SARS-CoV-2 cytokine storm, PARP14’s role in the interferon response to aid in clearing the virus, and the relationship between the deMARylating activity of the coronavirus macrodomain and PARP14’s antiviral activity. Further investigation of these hypotheses may give us a better understanding of the host immunopathological response to SARS-CoV-2 and aid in our future design of vaccines and treatments. PARP14 inhibitors may be clinically useful in the treatment of cancers and allergic airway diseases, but for coronaviruses the development of selective PARP14 inhibitors is more likely to be of use in furthering our understanding of the immunopathological response. Finally, we emphasize again that there needs to be more evidence-based data to support the potential role of PARP14 in SARS-CoV-2 infection. Use and continual development of selective PARP14 inhibitors will help to answer some of these hypotheses, and hopefully will aid us in designing better treatments and reducing the disease burden of COVID-19.

**Executive summary**

**SARS-CoV-2 infection**

- Severe acute respiratory syndrome coronavirus 2 (SARS-CoV-2) is the causative virus for the disease COVID-19.
- The SARS-CoV-2 macrodomain is shown to suppress the early interferon and proinflammatory cytokine response.
- When untreated, disease progression is closely linked to the systemic inflammatory response syndrome.

**PARP14’s potential role in SARS-CoV-2 infection**

- There are suggestions that PARP14’s role in the inflammatory response can help to counteract the Th1:Th2 cytokine ratio imbalance caused by SARS-CoV-2.
- PARP14 stimulation of IFN-1 may aid in activating the host defense to SARS-CoV-2 infection.

**PARP14 inhibitors**

- There are no clinically available PARP14 inhibitors.
- Currently the most potent PARP14 inhibitor is **H10** as proposed by Pen *et al*., which inhibits PARP14 with an IC_50_ of 0.49 ± 0.07 μM *in vitro*.
